# Confrontational scavenging as a possible source for language and cooperation

**DOI:** 10.1186/1471-2148-11-261

**Published:** 2011-09-20

**Authors:** Derek Bickerton, Eörs Szathmáry

**Affiliations:** 1 Department of Linguistics, University of Hawaii at Manoa, 1890 East-West Road, Honolulu, Hawaii 96822, USA; 2 Institute of Biology, Eötvös University, 1 c Pázmány Péter, H-1117 Budapest, Hungary; 3 Parmenides Centre for the Study of Thinking, Kirchplatz 1, D-82049 Pullach/Munich, Germany; 4 Collegium Budapest (Institute for Advanced Study), Hungary

## Abstract

The emergence of language and the high degree of cooperation found among humans seems to require more than a straightforward enhancement of primate traits. Some triggering episode unique to human ancestors was likely necessary. Here it is argued that confrontational scavenging was such an episode. Arguments for and against an established confrontational scavenging niche are discussed, as well as the probable effects of such a niche on language and co-operation. Finally, several possible directions for future research are suggested.

## Introduction

Language and cooperation form perhaps the most distinctive features of the human species. That language is unique to humans is almost universally acknowledged, even by those on opposite sides of the nativist/empiricist debate [1,2]. While closest relatives of humans have been shown to have a capacity, under intensive training, for rudimentary forms of language [3], there is no indication that this capacity would ever have developed in the wild. Cooperation on the scale practised by humans is found elsewhere only among the hymenoptera, although there the basis (kin selection) is quite different from that of human cooperation [4]. It would be remarkable enough if any species, in the 4-7 my (million-year) time-span suggested for human evolution, had developed just one of these traits. That each developed independently in the same species, with its own separate evolutionary history, seems unlikely.

Could co-operation have led to language, or vice versa? There are problems with either solution. A "language-first" model faces the difficulty that language presupposes a level of trust unlikely to exist given the conniving and deceit found among nonhuman primates [5]. Why would anyone believe verbal utterances, given that words are such "cheap signals" [6], and how, if no-one believed them, could language have taken root? However, a "cooperation-first" model faces an equal difficulty in that most evolutionary studies of human cooperation assume the existence of communal norms and the punishment of infractors [7-9]. It remains unclear how such norms could have been established without any kind of language.

The issues are further complicated by the fact that neither the evolution of language nor the evolution of co-operations is as yet well understood. There is still no consensus as to how language originated [10,11], while even recent work on the origins of co-operation. (typically treated as some mix of inclusive fitness, reciprocal altruism and group selection with other factors) [12,13] fails to offer an entirely convincing account of the differences in both degree and kind of cooperation that distinguish humans from apes. Most accounts of both language and cooperation invoke enhancement of, and interaction between, factors at least rudimentarily present in other species, even though, as niche construction theory [14] has recently emphasized, novel and unique traits from beaver dams to bat echolocation have typically originated through adaptation to some new and highly specific niche.

This is not to diminish the vital roles played by these pre-existing factors and the equally important roles they would play once language and cooperation had begun to develop. Clearly, such factors form necessary prerequisites for both language and cooperation, but were they sufficient? If they were, it remains a mystery why language and cooperation are hyper-developed in one species, while in the most closely related species they are either completely absent (language) or remain minimal (cooperation). The emergence of unique novel behaviours would appear to necessitate some equally unique triggering mechanism, such as entering a new niche that demanded such behaviours if the problems that niche presented were to be solved. In this paper we evaluate and strengthen the suggestion [15] that the confrontational scavenging scenario can resolve the issues.

## The case for a confrontational scavenging niche

Evidence exists that just such a niche may have been occupied by human ancestors starting around 2 mya and lasting perhaps several hundred thousand years. That niche was confrontational scavenging, sometimes called "power scavenging" [16]. However, no-one has yet claimed confrontational scavenging as a fully developed niche, and some have even denied that it could be one (see Section 3 below). Accordingly, before discussing possible consequences for human behaviour of a confrontational scavenging niche, the case for the existence of such a niche must be established. Although much of the evidence is of necessity indirect and inferential, it shows substantial convergence from diverse areas, including ecological opportunity, technology, sequences of bone markings, optimal foraging theory, and behavioural and physiological changes affecting *Homo *around 2 mya.

### Ecological opportunity

In the late Pliocene the East African climate became considerably drier and more variable, giving rise to large areas of savannah and many new species including herbivorous megafauna [17]. Carcasses of such megafauna offered immense quantities of meat. However, that meat could not be accessed immediately, because the toughness of megafauna hides made them impossible for mammalian teeth to breach until build-up of interior gases split them [18]. This left a window of at least several hours that could be exploited by any species capable of penetrating hides. Note that while megafauna carcasses were increasing in number, climate change was sharply reducing the staple primate diet of fruit and nuts.

Did such carcasses represent "rare bonanzas", as some sources have suggested [19], or were they common enough to have offered a (relatively) regular food source? There are no reliable estimates of prehistoric megafauna populations, so the best we can do is extrapolate from existing populations. Recent studies of elephant populations in two national parks [20,21] agree on a density of 0.3 per km^2^, but both sources note that in earlier decades populations were close to 1.0 per km^2^. For sustainable population density in a terrain similar to that of the climax-savannah period, an even higher density has been suggested [22]. Since elephants live on average 70 years, an area of 1,000 square kilometres--not an unreasonable annual range for *Homo erectus*--should yield on average a dead elephant at least every month. Moreover, elephant ancestors would have been far from the only megafauna available.

### Technology

Both the Oldowan and Acheulean industries provided flakes as a by-product of hand-axe and hammer-stone manufacture. It has been experimentally demonstrated that flake tools will quite easily cut through the hides of elephants, while Acheulean hand-axes are well adapted for subsequent butchery [18].

Hand-axes could have had a dual function in confrontational scavenging, since the latter would inevitably involve hostile interactions with competing scavengers (including predators larger and fiercer than their modern equivalents). Hand-axes have aerodynamic properties that would have made them useful projectiles [23]; scepticism about their effectiveness [24] relates to hunting rather than scavenging, where deterrence rather than killing or capture would be the goal. With flakes and hand-axes at their disposal, hominins had all the tools they needed to practice effective confrontational scavenging.

### Sequence of bone markings

The sequence of tool- and tooth-marks on bones can be read to infer priority of access to a carcass. If cut marks are superimposed on tooth marks (as is mostly the case before 2 mya) the carcass has been accessed by hominins only after other species have accessed it. If, however, tooth-marks are superimposed on cut-marks, human ancestors must have been the first to access the carcass. Priority of access could, of course, have resulted from either confrontational scavenging or hunting. However, hunting large animals requires adequate weapons, and there is no indication that contemporary hominins possessed these. A number of sources [19,25-27] indicate that subsequent to 2 mya this second sequence of bone markings occurred with increasing frequency. This could happen only if the hominins involved had been consistently accessing carcasses before other scavengers.

### Optimal foraging theory

Optimal foraging theory [28] proposes that a species will choose, out of available foods, those yielding the highest calorific gain relative to energy expended in obtaining them. Meat from megafauna carcasses offered the highest calorific yield available for any savannah-dwelling species (bone-marrow might be richer ounce for ounce, but quantities were far smaller). Engaging in confrontational scavenging does not preclude bone-marrow extraction; both might have been practised. However, since Oldowan technology is adequate for marrow-extraction but much less suited to confrontational scavenging than Acheulian technology, one plausible scenario would be a speciation event in which a hand-axe-wielding *erectus *split off from a bone-cracking *habilis*. Once located, exploiting a megafauna carcass took little time and energy relative to the nutritional yield. While confrontational scavenging was more dangerous than other foraging methods, risks could be reduced by numbers of participants and aimed throwing.

### Behavioural and physiological changes

The evolution of human scavenging falls into two phases: catchment scavenging and territory scavenging [29]. Catchment scavenging involved working within a restricted area where natural resources were plentiful, while territory scavenging involved ranging over much wider areas with relatively little attention paid to resource richness. The shift in strategies occurred, again, around 2 mya, and is consistent with a change from passive scavenging of mainly bones and discarded meat scraps to confrontational scavenging of entire carcasses, the location and timing of which were unpredictable. It is not necessary to claim that hominins became obligate confrontational scavengers (few if any animals are), only that confrontational scavenging was added to the arsenal of hominin behaviours as the currently most favoured strategy.

Given the 2 mya boundary between catchment and territory scavenging, we may (very roughly) associate the former with *Homo habilis *and the latter with *Homo erectus*. The two species show considerable physiological differences [30,31]: *erectus *was larger and taller than *habilis*, with a more rangy build, legs better adapted for rapidly covering long differences, and a considerably greater brain size. Were these changes adaptations for endurance hunting [32] or scavenging [33]? Since endurance hunting would have separated one or a very few individuals from the rest of the group, exposing them to predation at a time before there were spears or arrows for self-defence, confrontational scavenging (which might have involved covering long distances, but in larger groups) seems a more plausible selective pressure for the suite of behavioral and physiological characteristics found in *erectus*.

## Arguments against confrontational scavenging

Although, as shown above, a wide range of data is consistent with the confrontational scavenging hypothesis, a number of scholars have suggested that the practice cannot have played any significant role in human development. For instance, Dusseldorp [34] argues that "Since scavenging is a competitive niche requiring specialization, it was considered unlikely that hominins ever relied on this strategy." However, niche construction theory has shown that specialization does not precede exploitation of a niche--rather, new-niche occupation by initially unspecialized species is precisely what drives specialization [14].

The fact that scavenging behaviour is rare or non-existent among other primates is often taken as evidence against scavenging by human ancestors [35]. However, this argument is based on the assumption that all human behaviour is grounded in ape behaviour, and that straight-line evolution from great apes is an indisputable fact. There seems little empirical evidence for such assumptions. Behaviour is determined at least as much by environmental and ecological considerations as by genetic factors; consider for instance the fact that the hyrax, the elephant and the manatee are more closely related to one another than they are to other species [36]. If human ancestors found that scavenging was necessary for subsistence, it is unlikely that great-ape genes would have deterred them.

The belief that megafauna carcass finds were rare events, discussed in Section 2.1 above, seems to rest on the assumption that our ancestors would have been limited to carcasses that, to their surprise perhaps, they happened to stumble upon. It is much likelier that organisms whose brain sizes often fell within the human range would have taken a proactive approach, perhaps even tracking and following herds as was done by humans in the Upper Paleolithic. While the returns of such a strategy might not always have sufficed for subsistence, temporary reversion to gathering, bone scavenging and opportunistic hunting was always a possibility.

Finally, Geist [35] asks, "If scavenging was important, how did it lead to the adaptations characteristic of *Homo?*" The remainder of this paper seeks to answer this precise question, giving substance to the caveat that ends an earlier sentence by the same author: "Scavenging is not part of a sufficient explanation of human adaptations--*at least not yet*" (emphasis added).

## Why confrontational scavenging required atypical primate behaviours

To access megafauna carcasses in the face of severe competition required that human ancestors communicate in ways no other primate had done, and cooperate to a degree unknown among other primates.

There are no reliable estimates for human band size circa 2 mya. Due to the sparseness of food supplies under savanna conditions, as well as other factors that limit band size even in modern foraging populations [37], it seems likeliest that bands of < 40 individuals would have followed the common primate behaviour of fission-fusion foraging, dividing into smaller groups (but not too small to protect against predation) by day to cover the maximum area, and meeting in refugia at night. To simultaneously butcher a carcass and drive off competitive scavengers required more numbers than such smaller groups could provide, necessitating the active co-operation of a large majority in each band.

To recruit adequate numbers, two critical acts had to be performed. The carcass finders had to communicate information that lay far outside the sensory range of potential recruits, and they had to convince those recruits to abandon whatever they were doing and travel to a perhaps quite distant site for an invisible goal.

### The communication problem

The vast majority of animal communication systems, including all other primate systems, are unable to give information about anything that falls outside the potential sensory range of recipients. Exceptions are the systems of eusocial species (ants [38], bees [39]...) that share a feature otherwise unique to human language, displacement, making it possible to refer to events remote in space and/or time. It is noteworthy that ants and bees also regularly forage food sources (pollen in the case of bees, larger dead organisms in the case of ants) that are too large and/or too transient for one or a few individuals to fully exploit; these are precisely the circumstances that require recruitment. The location of such sources, which may lie some distance (in the case of bees, miles) away, is signalled by stereotyped dances (bees) or chemical or behavioural [40] means (ants). In other words, animals are limited only by their phenotypes in the means of signalling they adopt.

Band members who had located a carcass would have had to use sounds, gestures or mimicry to inform potential recruits of what they had found. The modality, as the hymenopteran examples suggest, is unimportant--whatever worked would have been chosen, what mattered was displacement itself. Reference to absent entities is the first step towards symbolism, the capacity that underlies all of human cognition including language [41]. While it is true that in ants and bees, displacement never moved further towards true symbolism, its consequences would have been far more widespread in a species with 4 × 10^4 ^more brain cells and a large suite of prerequisites for language.

### The cooperation problem

It is one thing to convey information--quite another to get other individuals to act on that information.

Primates are not known for their cooperativeness. To the contrary, they are highly competitive and willing to deceive one another in pursuit of individual goals [5]. They cooperate only to the extent that this furthers the latter, and only on the basis of things immediately apparent to them. This type of behaviour has been called "competitive cooperation", as opposed to "collaborative cooperation" where "the resource is not manifest, but mainly imagined" [42] (the latter being typical of human cooperation).

Thus, even assuming that communication was successful (i.e. recipients realized that a megafauna carcass was available at location X), it does not follow that recipients would automatically co-operate. Those who did not go for the carcass would suffer a penalty in terms of less food (unless dependent and needy); for such punishment only memory and individual recognition were needed. Boyd et al. considered the origin of coordinated punishment. The authors observe that "A complete account of the evolution of cooperation must explain how punishing strategies can increase when rare." [[43], p. 620]. Here they wouldn't have been rare even at the beginning. Imagine a situation in which three out of four subgroups of the band co-operate but the fourth doesn't. As a direct result, scavengers drive the band from the carcass, and three-fourths of the band go hungry. It does not stretch the imagination to ponder the consequences of such defection. The key point in the scenario is that all members of the group must cooperate simultaneously if a goal advantageous to all is to be achieved. Individuals who nonetheless wish to avoid physical danger during the action would be immediately noted and punished in a coordinated way by the same group that had already cooperated in the scavenging. These considerations do not exclude the possibility that reputation-based punishment may have later contributed to the extension of cooperation [44,45], but we also note that the cited models concern pairwise interactions only. Some of the tools used in scavenging may have been used as projectiles for coordinated punishment [46] of defectors after the event, but we feel that such use at the stage discussed may have been rather casual.

One way to determine the probabilities here would be to look at temporal profiles of contemporary megafauna carcass sites. Surprisingly there seems to be little if any literature on this topic. Available evidence comes mainly from wild-life photographers, and suggests considerable variability at such sites. In one account, a hippopotamus was being consumed by nineteen lions, although how long the carcass had been available is not known [47]. In another, however, an elephant that "must have died within the last day or two" was found to be "surprisingly intact", and attended only by a few jackals and a number of vultures [48]; it was only some days later that lions appeared.

Empirically it is known that in the case of cooperative hunting not everybody cooperates (there is always a fraction of laggards), but even more interesting, it seem that in lions [49] and at least in some hunter-gatherer groups [50,51] such laggards are *not *punished. Two remarks are in order. First, we do not know whether this finding generalizes to all hunter-gatherers, even less whether it applied to ancient scavengers. Second, as we shall see shortly, in similar games there can be stable coexistence of cooperators and defectors even without punishment.

### Supporting models

Though an adequate model involving all important features (communication, cooperative scavenging, group competition and punishment) does not exist in the literature, we call attention to the fact that non-linear *N-*person public good games (reviewed in [52]) offer very encouraging results already in the right direction. In these models cooperators pay a cost and contribute to a public good that, in the simplest case, can be consumed by all members. Cooperators pay a fixed per capita cost and benefits are shared. Non-linearity means that fitness increases non-linearly with the frequency of cooperators. For the scavenging scenario the "teamwork dilemma" [53] is the most relevant where it holds that out of a group of *N *players at least *k *must cooperate to produce the public good, thus *k *is the threshold value. A general outcome of these games is that unless the costs are prohibitively high, there is the possibility of locally stable coexistence even in a well-mixed population with random assortment into potentially cooperating groups [52]. Remarkably, the sharp threshold can be replaced practically with any sigmoid benefit function (increasing and diminishing returns for low and high numbers of actual cooperators, respectively). Punishment is not necessary for the existence of the cooperative equilibrium.

Boza and Számadó have developed these models further in order to elucidate the effects of multilevel selection (with or without local population structure) [54]. It was assumed that during group competition a group with a higher average payoff replaces a randomly chosen group with lower average fitness. Interestingly, they found that group selection does not lead to the fixation of the cooperative form either, but it brings the frequency of cooperators closer to the optimum number for the group (intuitively, if *k *cooperators are sufficient for the teamwork, the involvement of more agents is wasteful). Local spatial structure promotes cooperation in the model as it maintains a high level of cooperation even with high costs for both hunting and defence. It is even possible for cooperators to invade when rare, despite the positive costs to cooperation.

### Consequences for language and cooperation

Of course the processes described above, even if successfully completed, could not, in and of themselves, have led to anything remotely approaching the levels of language or cooperation that characterize modern humans. What they could do, in a species with at least the cognitive capacities of a great ape, was kick-start two autocatalytic processes that could then join in a co-evolutionary spiral leading eventually to modern humans.

With regard to the first, communication, all that confrontational scavenging made available was an enhanced system of communication--a typical animal communication system (ACS) plus displacement. But displacement would have served as the wedge that broke the walls of the here-and-now that circumscribe almost all ACSs (including all ACSs of other neurologically-complex organisms), potentially allowing free reference to anything in the world, past or future, real or imagined. While at first this enhanced ACS might have been used solely for foraging, its utility in other spheres--pedagogy, planning, manipulation of others, and much more--would surely have become apparent, allowing its uses to extend, and its resources to become richer, as fast as the neurobiological changes necessary for full language could develop.

With regard to the second, cooperation, successful execution of confrontational scavenging would have immediately yielded tangible proof (in terms of a steady supply of high-quality nutriments) of the benefits of cooperation. Once it had been established, in one sphere at least, that cooperative strategies yielded better results than competitive strategies, such strategies would surely have spread to other domains. Increases in ways of cooperating would then have selected for genes that either favoured cooperation or suppressed overly competitive behaviours.

As Szathmáry and Számadó remark: "The evolution of language probably occurred in concert with the evolution of many of the other traits we associate with being human, such as the ability to fashion tools or a strong propensity to learn. If this is true, it suggests that we shouldn't be trying to understand one characteristically human trait in isolation from the others." [55]. Consonant with this view, the notion of a 'human-specific adaptive suite' entails a number of synergistic traits, including eminently language and cooperation, where selection on one would have accelerated the evolution of others [56]. The more cooperation, the more language was needed to practice it; the more language, the more avenues for cooperation opened up. All the factors previously invoked as directly selective of language and/or cooperation, from theory of mind to group selection, would have come into play. But as suggested in Section 1, language and cooperation on a human scale are so far from primate norms that the mere presence of potential preadaptations could not alone have precipitated them. They required a trigger of some kind, one unique to immediate human ancestors, and so far as is presently known confrontational scavenging was the only activity practiced by those ancestors that contained all the necessary components for such a trigger.

An analysis of the selective scenarios for language [57] concluded that none of them could account for all the criteria established for evaluation: honesty, groundedness, power of generalization and uniqueness. It strikes us that the cooperative scavenging scenario, for the first time in the literature, meets all these criteria.

## Future research

The foregoing should serve as a prima facie case for regarding confrontational scavenging as a well-defined niche and possibly crucial in the emergence of human cooperation and language. However, much needs to be done in several areas of research before more definite conclusions can be reached.

Perhaps the most fundamental issue concerns how species with novel traits originate. The vast majority of explanations for how language and cooperation arose (a) fail to make specific reference to any detailed account of human evolution and (b) adopt multifactorial explanations of a kind seldom if ever invoked when other species are discussed [7-13]. More fine-grained studies of speciation are needed to determine what kinds of selective pressure cause such events.

A better understanding of the ecology of 2 mya, and how human ancestors fitted into it, is also required. Many parts of the jigsaw are to be found in this area. We need at least a rough estimate of population figures for a wide variety of species and we need a better understanding of hominin foraging patterns, including daily and annual ranges and band size. A vexing problem lies in the fact that territory scavenging entails deposits of bones that are widely and thinly scattered, making it difficult to determine the full scope of the role confrontational scavenging played in overall foraging patterns.

The suggested use of hand-axes as projectiles in the deterrence of rival scavengers should also be tested to resolve as far as possible disagreements over the functionality of this procedure. Careful statistical studies of microwear patterns and spatial distribution of hand-axes would help us determine their function(s).

Among the most potentially revealing of the studies suggested by the confrontational-scavenging hypothesis would be those involving species that faced a similar ecological problem: the need to practice recruitment in order to exploit large, unpredictable and transient food sources, with or without the complication of competition. Surprisingly few species seem to be faced with this problem; apart from bees and ants, the only case reported so far involves ravens [58]. Juvenile ravens are driven away from carcasses (in winter, almost the only form of nutrition) by mature mated pairs, but (apparently by exchanging information in nightly roosts) the former often manage to recruit sufficient numbers to drive the latter off. Here one finds competition within rather than between species, but the principles involved are the same. Whether ravens have indeed mastered displacement, and if so how they accomplish it, is currently under investigation [59].

There may, of course, be other species that face similar problems. Such species too would need to be studied from a comparative perspective. Even the existence of the few we know raises fascinating questions concerning the extent to which, given details of any ecological problem, we can predict how a species will solve it, and whether successful solutions are repeated regardless of phylogenetic distance between the species concerned. And even if the confrontational scavenging hypothesis fails to find support, the research it suggests should shed much light on both the history of our species and evolutionary processes in general. The aim is, as with other major transitions in evolution [60], to put forward a scenario about which we can all agree that this is how it could have happened, without necessarily ever being able to say that this was exactly how it did happen.

Our account is a logical analysis of a promising scenario. The phenomena we consider are dynamically complex, and in the absence of direct experimentation, strongly call for a modelling approach. In the next paper we demonstrate how communication and cooperation can together triumph in a population model (Szathmáry and Bickerton, forthcoming).

## Authors' contributions

DB conceived the paper, wrote the first draft. ES has contributed the discussion of models. Both authors read and approved the final manuscript.
